# Akute genitale Ulzerationen nach COVID-19-Infektion und Coronaimpfung

**DOI:** 10.1007/s10304-022-00485-z

**Published:** 2022-12-15

**Authors:** Bettina Böttcher, Sigrid Schmidl-Amann, Bettina Toth

**Affiliations:** 1 Klinik für Gyn. Endokrinologie und Reproduktionsmedizin, Medizinische Universität Innsbruck, Anichstr. 35, 6020 Innsbruck, Österreich; 2 St. Pölten, Österreich

## Einleitung

Akute genitale Ulzerationen bei Jugendlichen ohne Geschlechtsverkehr sind selten, können aber sehr schmerzhaft sein und die Patientinnen körperlich und psychisch stark belasten. Am häufigsten werden sie durch das Herpes-simplex-Virus (HSV) verursacht oder stehen in Zusammenhang mit anderen Virusinfektionen wie dem Epstein-Barr-Virus (EBV) oder dem Zytomegalievirus (CMV; [1, 2]). Akute Genitalulzera bei Mädchen ohne Geschlechtsverkehr bzw. ohne bekannte Ätiologie wurden früher als Lipschütz-Ulzera bezeichnet [2, 3]. Die Differenzialdiagnosen umfassen Morbus Behçet, Morbus Crohn, Arzneimittelreaktionen oder sexuell übertragbare Krankheiten wie Syphilis oder HIV [4]. Die zugrunde liegende Pathogenese ist bisher nicht abschließend geklärt, eine dysregulierte Immunreaktion ist anzunehmen.

In der wissenschaftlichen Literatur und den Medien wurden mögliche immunologische Nebenwirkungen des Coronaimpfstoffs kontrovers diskutiert. Mehrere Berichte über akute genitale Ulzerationen bei Adoleszentinnen nach einer Infektion mit SARS-CoV‑2 oder nach einer Coronaimpfung wurden bereits veröffentlicht [5–7]. Um diese Diskussion zu ergänzen, stellen wir zwei Fälle vor, in denen akute genitale Ulzerationen bei Mädchen nach einer SARS-CoV-2-Infektion und nach einer Coronaimpfung aufgetreten sind.

## Anamnese

### Fall 1

Ein 13-jähriges Mädchen stellte sich eine Woche nach der zweiten Coronaimpfung mit einer genitalen Ulzeration vor. Ihre Eigen- und Familienanamnese waren unauffällig. Sie hatte ihre Menarche im Alter von 12 Jahren und verneinte jegliche sexuelle Aktivität. Drei Wochen zuvor hatte sie die erste Impfung ohne Nebenwirkungen erhalten. Nach der zweiten Impfung bekam sie zwei Tage lang Fieber, gefolgt von einer Schwellung der rechten Vulvalippe. In den folgenden drei Tagen litt sie unter zunehmenden Schmerzen und stellte sich bei ihrer Frauenärztin vor. Sie konnte sich nicht mehr hinsetzen, hatte massive Schmerzen beim Wasserlassen und konnte nur noch lockere Hosen tragen.

### Fall 2

Bei einem 11-jährigen Mädchen wurde zufällig eine SARS-CoV-2-Infektion festgestellt. Symptome lagen zunächst nicht vor. Sie hatte eine unauffällige Vorgeschichte und noch keine Menarche. Eine Woche nach der Diagnose traten Kopfschmerzen, Fieber und Dysurie auf. Vier Tage nach Beginn der Symptome wurde das Mädchen mit einer Harnwegsinfektion ins Krankenhaus eingeliefert und erhielt eine Antibiotikatherapie mit Ampicillin/Sulbactam. Im Verlauf berichtete sie über brennende Schmerzen an der rechten Vulva. Aufgrund der Verdachtsdiagnose eines Herpes genitalis wurde eine Behandlung mit Valaciclovir begonnen.

## Befund

### Fall 1

**Figure 1:**
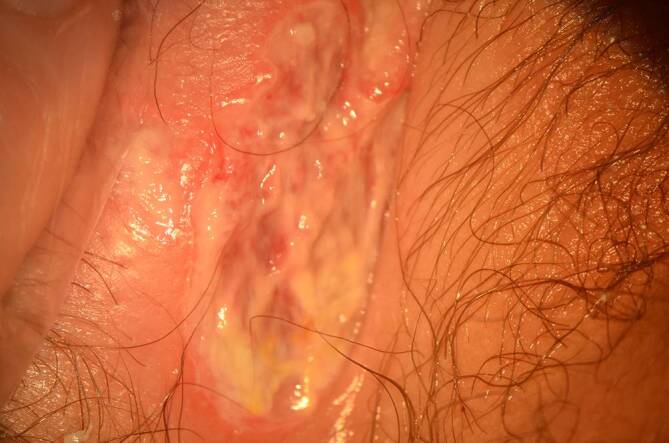

Bei der Untersuchung wurde eine aphthöse Ulzeration über die gesamte Außenseite der rechten großen Vulvalippe mit gelbem fibrinösem Exsudat festgestellt (Abb. 1 und 2). Im Vergleich zur linken Vulva wies die rechte Seite ein massives Ödem auf. Eine inguinale Lymphadenopathie lag nicht vor.

**Figure 2:**
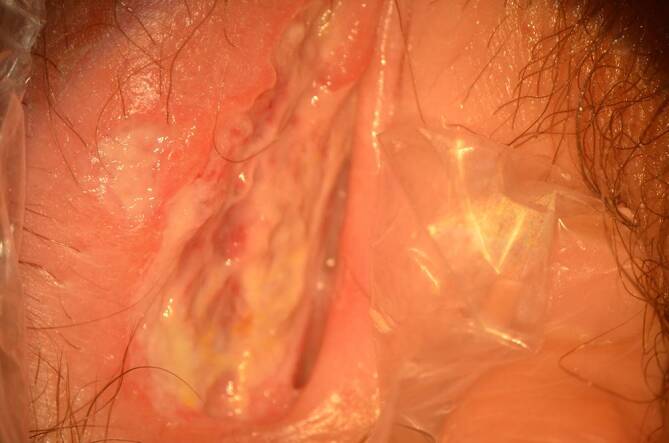

### Fall 2

**Figure 3:**
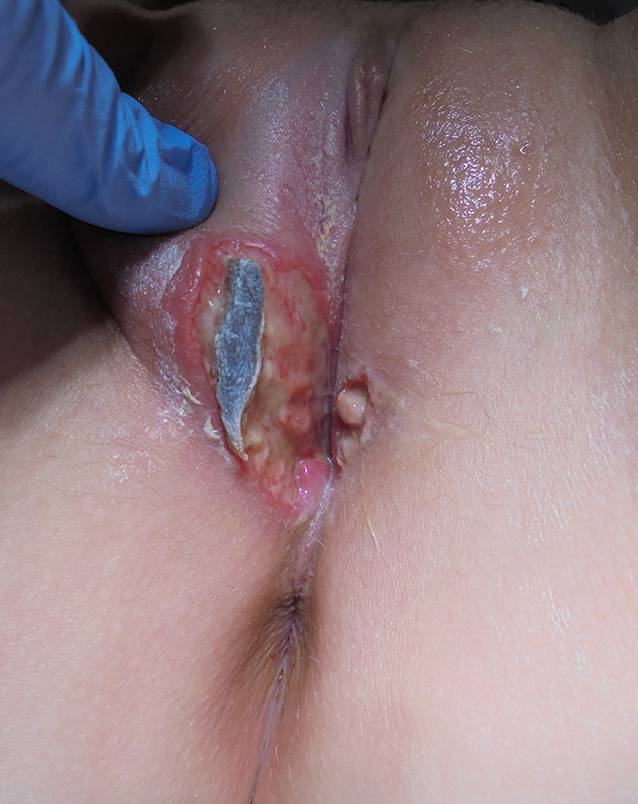

Die Ulzerationen vergrößerten sich. Zusätzlich kam es zur Schwellung der rechten Vulva. Nach der Entlassung aus dem Krankenhaus stellte sich das Mädchen bei ihrer Frauenärztin vor: An der rechten großen Vulvalippe fand sich eine 4 × 3 cm große Ulzeration mit anhaftendem gelbem und grauem Exsudat, einem grauen Schorf und einem scharf abgegrenzten ödematösen Rand (Abb. 3). Die Ulzeration auf der linken Seite war kleiner und betrug 1 × 1 cm (Abb. 3). Es gab keine Anzeichen einer Lymphadenopathie. Das Mädchen litt unter starken Schmerzen, vor allem beim Sitzen und Wasserlassen.

## Diagnose

Aufgrund des klinischen Bilds wurden bei beiden Mädchen akute genitale Ulzerationen diagnostiziert.

## Therapie und Verlauf

### Fall 1

**Figure 4:**
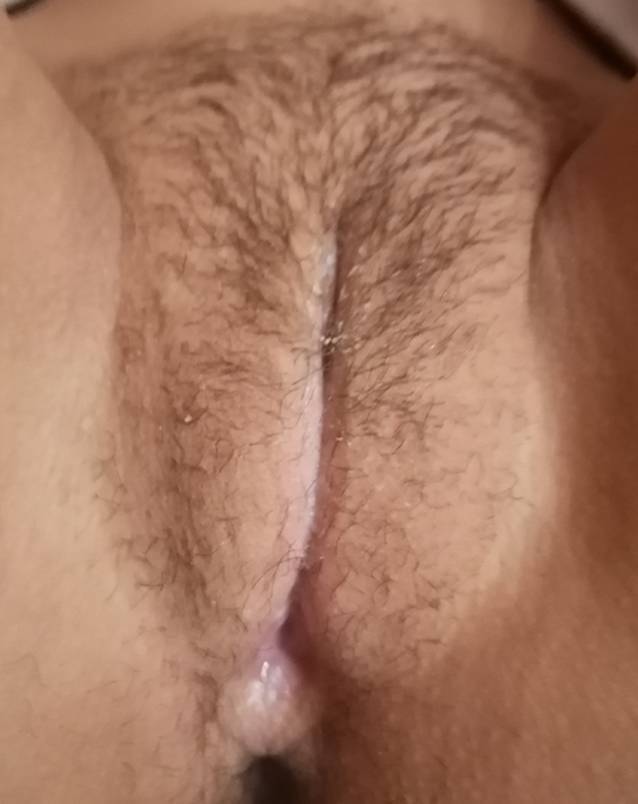

Die Patientin erhielt eine Schmerzmedikation, Betaisodona-Sitzbäder und eine jodhaltige Salbe. Einen Tag später stellte sie sich erneut vor, weil sie noch Schmerzen beim Wasserlassen hatte. Die Läsion zeigte bereits Anzeichen einer Granulation. Drei Tage später ließen die Schmerzen nach und verschwanden fünf weitere Tage später vollständig. Der Heilungsprozess war nach 14 Tagen narbenlos abgeschlossen (Abb. 4).

### Fall 2

**Figure 5:**
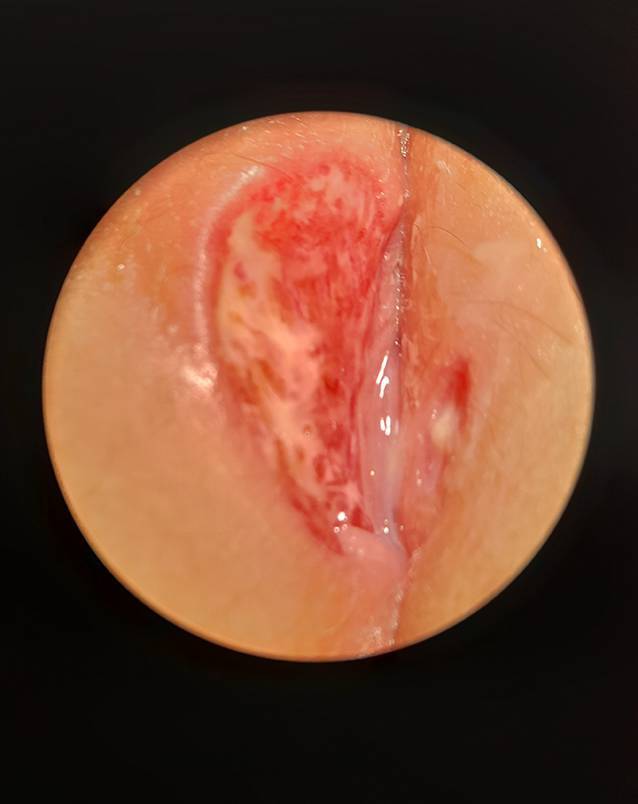

Die Therapie mit Antibiotika und Schmerzmedikation wurde fortgesetzt. Sitzbäder mit Betaisodona und eine Lokalanästhesie vor dem Wasserlassen wurden begonnen. Nach drei Tagen ließen die Schmerzen nach, die Dysurie war noch fortbestehend. Bei der Untersuchung zeigten die Ulzerationen sich kleiner und das Gewebe begann zu granulieren (Abb. 5). Der fibrinöse Belag war rückläufig. Nach einer weiteren Woche war die Patientin schmerzfrei und eine weitere Woche später zeigte sich ein narbenfreier Lokalbefund.

## Diskussion

Diese beiden Fallberichte über akute genitale Ulzerationen bei Adoleszentinnen nach einer Infektion mit COVID-19 bzw. nach einer Coronaimpfung dagegen deuten auf einen möglichen Zusammenhang hin. Beide Patientinnen wiesen typische Anzeichen für akute genitale Ulzerationen wie massive Schmerzen im Genitalbereich, ödematöse Schwellungen und große (> 1 cm), flache und scharf begrenzte Ulzerationen mit ausgedehnten fibrinösen Exsudaten auf. Zudem berichteten beide Patientinnen über unspezifische virale Prodromi, die ebenfalls typisch für akute genitale Ulzerationen sein können [1, 5].

Es wird vermutet, dass die Pathogenese durch eine unspezifische Entzündungsreaktion getriggert wird, die durch verschiedene Virusinfektionen (EBV, CMV, Influenza, Syphilis, Salmonellen u. a.) ausgelöst werden kann [2, 3, 7]. Am häufigsten wird ein Zusammenhang mit einer Primärinfektion mit EBV beobachtet [3]. Das HSV sollte differenzialdiagnostisch ausgeschlossen werden. Dieser Pathomechanismus könnte auch im Falle einer Infektion mit SARS-CoV‑2 zutreffen, da ebenso eine systemische Entzündungsreaktion entsteht. Vulväre Ulzerationen nach einer Coronaimpfung sind selten [5]. Dennoch wurden Nebenwirkungen wie Blasen und Ulzerationen an der Mundschleimhaut nach Verabreichung des SARS-CoV-2-Impfstoffs bei 10–13 % der geimpften deutschen, tschechischen, türkischen und slowakischen Mitarbeiter:innen im Gesundheitswesen in vier verschiedenen Studien berichtet [8–10].

Die in den vorliegenden Fallberichten beschriebenen Patientinnen wurden mit Lokalanästhetika, Salben sowie Sitzbädern und Schmerzmedikation behandelt. Eine wegen einer Harnwegsinfektion begonnene Antibiotikabehandlung wurde fortgesetzt. Im Allgemeinen werden akute Genitalulzera bei sexuell nicht aktiven Mädchen zur Symptomlinderung mit oralen Schmerzmitteln behandelt [1–3]. Sitzbäder, orale und intravenöse Antibiotika und die Gabe von Kortikosteroiden ergänzen die Behandlungsmöglichkeiten. Topische Steroide wie z. B. Clobetasol-Salbe (0,05 %) können die dermatologischen Symptome verbessern, eine systemische Kortikosteroidbehandlung bei akuten Genitalulzera wird in der Literatur hingegen kontrovers diskutiert [1, 7, 11]. Orale Kortikosteroide können bei rezidivierenden akuten Genitalulzera oder bei Patientinnen, die nicht ausreichend auf eine lokale Therapie ansprechen, indiziert sein [2]. In den meisten Fällen sind Biopsien für die Diagnose nicht unbedingt erforderlich und sollten nach Möglichkeit vermieden werden, insbesondere bei Adoleszentinnen.

Es besteht die Hypothese, dass nichtsteroidale Antirheumatika (NSAR) mit der Entwicklung von oralen und vulvären Aphten in Verbindung gebracht werden können [1, 12]. Obwohl kein pathophysiologischer Mechanismus identifiziert und kein Zusammenhang nachgewiesen werden konnte, wird empfohlen, NSAR in diesen Situationen zu vermeiden [1]. Stattdessen sollten Schmerzmittel mit Paracetamol in Kombination mit lokalem Lidocaingel und Sitzbädern verabreicht werden [1]. Die meisten Mädchen können ambulant behandelt werden. Eine stationäre Aufnahme kann erforderlich sein, wenn das Wasserlassen zu schmerzhaft ist und eine Blasendrainage erforderlich wird oder zur Schmerzkontrolle [1].

Die mediane Zeit bis zur vollständigen Heilung der akuten genitalen Ulzerationen beträgt etwa 17–21 Tage [2–4]. Kontrollen bis zum Abklingen der Schmerzen werden empfohlen [2].

## Fazit für die Praxis

Akute genitale Ulzerationen können zu starken Schmerzen und psychischer Belastung führen. Daher sollten diese Patientinnen eine ausreichende Schmerzmedikation und Lokaltherapie erhalten. Eine Biopsie zur Diagnosesicherung ist nur selten erforderlich. Die meisten akuten Genitalulzera heilen spontan ab, ohne dass es zu Narben, Rezidiven oder Folgeschäden kommt. Diese Fallberichte deuten auf eine Assoziation von akuten genitalen Ulzerationen bei Adoleszentinnen nach einer COVID-19-Infektion und nach einer Coronaimpfung hin, beweisen aber keinen kausalen Zusammenhang.
